# Effects of a Balanced Diet and Probiotics on Blood Biomarkers and Gut Microbiota in the Elderly: A Community-Based Intervention Study

**DOI:** 10.3390/nu17111933

**Published:** 2025-06-04

**Authors:** Junghyun Park, You-Suk Lee, Do-Kyung Lee, Juyong Hong, Seon-Joo Park, Byung Wook Lee, Sang Min Park, Hae-Jeung Lee

**Affiliations:** 1 Department of Food and Nutrition, College of BioNano Technology, Gachon University, Seongnam 13120, Republic of Korea; iwbstill@yahoo.com (J.P.); boknyu88@naver.com (D.-K.L.);; 2 Institute for Aging and Clinical Nutrition Research, Gachon University, Seongnam 13120, Republic of Korea; ysleeyun@gachon.ac.kr; 3 Department of Agricultural Biotechnology, College of Agriculture and Life Sciences, Seoul National University, Seoul 08826, Republic of Korea; hong92820@snu.ac.kr; 4 Pharmsville Co., Ltd., Seoul 07793, Republic of Korea; apple6@pharmsville.com (B.W.L.); plan1@pharmsville.com (S.M.P.); 5 Department of Health Sciences and Technology, GAIHST, Gachon University, Incheon 21999, Republic of Korea

**Keywords:** balanced diet, probiotics, aging, gut microbiota, biomarkers

## Abstract

Background/Objectives: Aging is characterized by dysregulation of the immune system. A balanced diet and probiotic intake can play significant roles in preventing aging-related chronic degenerative diseases and promoting immune function in the elderly. This community-based intervention study investigated the effects of an eight-week intervention involving a balanced diet with or without probiotics on nutritional parameters and gut microbiota composition in Korean elderly individuals. Methods: A total of 48 participants were enrolled and randomly allocated into two groups: 24 received a balanced diet alone, and 24 received a balanced diet combined with probiotics. Results: The balanced diet showed beneficial impacts on nutritional and inflammatory biomarkers, including fasting glucose, hemoglobin A1c (HbA1c), albumin, gamma-glutamyl transferase (γ-GT), total cholesterol, high-density lipoprotein cholesterol (HDL-C), immunoglobulin E (IgE), and gut microbiota, such as the *Bacteroidaceae* family and the genera *Prevotella* and *Faecalibacterium*. Additionally, providing probiotics alongside a balanced diet influenced the taxonomic profile and abundance of intestinal microbiota. Conclusions: Overall, the combination of a balanced diet and probiotics has beneficial effects on nutritional and inflammatory biomarkers as well as gut microbiota composition in elderly individuals. Future meticulously designed randomized controlled trials are required to further understand the health benefits and underlying mechanisms of balanced diets and probiotics in the Korean elderly.

## 1. Introduction

Rapid population aging has been considered a key factor contributing to rising healthcare expenditure in Korea [1], highlighting the urgent need to prevent and manage chronic degenerative diseases related to aging. Among various modifiable factors, diet plays a central role in maintaining overall health and mitigating age-related disease risks. In this context, a balanced diet that meets recommended nutrient intake levels is crucial for older adults, not only to prevent chronic conditions, but also to support general well-being.

The 2020 Dietary Reference Intakes for Koreans (2020 KDRIs), provided by the Korean Nutrition Society and the Ministry of Health and Welfare, recommends daily calorie intakes of 2000 kcal for men and 1600 kcal for women aged 65 and older, along with adequate macronutrient ratios (carbohydrates 55–60%, protein 7–20%, and fat 15–30%) and protein intake (60 g/day for men and 50 g/day for women) [2]. However, national survey data reveal that Korean elderly individuals overall tend to have insufficient energy and protein, calcium, potassium, riboflavin, and vitamin A intake [3]. These nutritional inadequacies are further exacerbated by age-related changes in oral function, such as chewing and swallowing difficulties, which often lead to avoidance of certain food textures and reduced food variety [4,5].

In parallel, aging also involves dysregulation of the immune system, characterized by increased levels of inflammatory biomarkers such as cytokines, and C-reactive protein (CRP), contributing to a state of chronic low-grade inflammation known as “inflammaging” [6,7]. Emerging evidence suggests that probiotics may offer immunomodulatory benefits by improving low-grade inflammation, counteracting immunosenescence, and supporting gastrointestinal (GI) health through the modulation of the gut microbiota and the gut–brain axis [6,7,8,9]. These effects, however, may differ by age groups, as elderly individuals exhibit distinct immune responses to probiotics due to age-related immunological changes.

Considering these interrelated factors, nutritional inadequacy, oral function decline, immunological aging, and the potential dietary and probiotic interventions, this study aimed to investigate the effect of an eight-week community-based intervention involving a balanced, elderly friendly diet with or without probiotics on nutritional parameters and gut microbiota composition in older Korean adults.

## 2. Materials and Methods

### 2.1. Participants

This study recruited healthy elderly individuals aged 65 years and older residing in Bundang-gu, Seongnam-si, Gyeonggi Province, through flyers, poster advertisements, local newspapers, and community centers for the elderly. Participant recruitment took place from July to September 2022. Individuals who agreed to participate in this community-based intervention study were enrolled. Exclusion criteria included a history of stroke, severe illness such as malignant tumor, difficulty swallowing, continuous probiotic intake within the past two months, dementia or other cognitive impairments, psychiatric history within the past six months, or severe food allergies. Before the study commenced, all participants received both written and verbal information about the study and provided informed consent. This study was approved by the Institutional Review Board of Gachon University (IRB No. 1044396-202205-HR-108-01).

### 2.2. Study Design and Intervention

A parallel, randomized, single-blind study was conducted over an eight-week period from September to November 2022 at Gachon University, South Korea. Participants were randomly assigned to one of two groups using randomization codes: (1) a balanced diet intake group (B-diet group) or (2) a balanced diet and probiotics intake group (B-diet + probiotics group).

The balanced diet, designed to be easy to swallow and digest, was provided by Hyodokook Corporation (Agape and Feeds Co., Ltd., Seongnam-si, Republic of Korea). The meals were formulated to meet the caloric and nutritional requirements (especially, protein, calcium, and vitamin D including other nutrients) of individuals aged 65 and older, based on the 2020 KDRIs established by the Korean Nutrition Society. The diet plan was developed by registered dietitians using the Smart Dietitian Management System, a specialized web-based diet planning software available at https://www.yori.co.kr/ (accessed between 1 October and 18 November 2022). The balanced meals were delivered in a lunch box format for both lunch and dinner daily. Each meal provided 670 kcal for men and 530 kcal for women, based on a total daily intake of 2000 kcal for men and 1600 kcal for women. Participants were instructed to maintain their usual diet for breakfast, the non-intervention meal, and received education from a registered dietitian on how to achieve a balanced overall diet in accordance with the Korean Dietary Reference Intakes (KDRIs).

Synbiotics, consisting of probiotics and prebiotics, were provided in the form of *Pharmsville gut health 365 synbiotics* (Pharmsville Co., Ltd., Seoul, Republic of Korea). Each dose contained 17 probiotic strains, consisting of Lactococcus lactis, Lactobacillus reuteri, and 15 other probiotic strains, combined with prebiotics in a proprietary formulation by Pharmsville. Participants in the B-diet + probiotics group consumed two sachets daily (5.5 g per sachet). Each sachet of the probiotic mix contained approximately 1 × 10^8^ CFU, corresponding to ~1.82 × 10^7^ CFU per gram. Participants were instructed to take them immediately after meals or within 30 min postprandially.

Participants in the B-diet group received only balanced meals for lunch and dinner, while those in the B-diet + probiotics group received both balanced meals and probiotics. Before and after the intervention, anthropometric measurements and blood pressure assessments were conducted. Evaluation of physical activity and functional ability and dietary intake assessments (via 24 h recall) were performed using structured questionnaires. Blood and fecal samples were collected for biomarker analysis and gut microbiota profiling. Due to the high prevalence of presbyopia and other visual impairments among elderly populations, which could affect their ability to read or understand survey questions, all surveys were administered through individual face-to-face interviews conducted by trained nutritionists.

To assess adherence to the intervention, a designated researcher monitored lunchbox deliveries, the return of empty lunchboxes, and probiotic consumption levels on a weekly basis via phone calls and social media (KakaoTalk, version 3.4.3.3219). Single blinding was maintained throughout the study period, ensuring that participants remained unaware of their group allocation until the study concluded.

### 2.3. Anthropometric Measures and General Characteristics

Anthropometric and blood pressure measurements were performed, including height, weight, and blood pressure. Body mass index (BMI) was calculated based on height and weight measurements. BMI classification was conducted according to the criteria established by the Korean Society for the Study of Obesity, categorizing individuals as follows: underweight (<18.5 kg/m^2^), normal weight (18.5~23.0 kg/m^2^), overweight (23.0~25.0 kg/m^2^), and obese (≥25.0 kg/m^2^).

General characteristics were assessed through a structured questionnaire, including information on educational attainment, stress levels, sleep duration, alcohol consumption, smoking habits, regular physical activity, and physical functionality. Physical functionality was evaluated using the Korean-Instrumental Activities of Daily Living (K-IADL) questionnaire [10]. This tool assesses participants’ ability to perform instrumental activities of daily living, including telephone use, shopping, meal preparation, household chores, laundry, transportation, medication management, and financial management. Each activity was rated on a 4-point scale, generating a composite score for K-IADL assessment.

### 2.4. Primary Outcomes

#### 2.4.1. Blood Markers of Nutritional Status and Inflammation

Fasting venous blood samples were collected before and after the intervention following an 8 h fasting period for blood biomarker analysis. The biomarkers measured included fasting blood glucose, glycated hemoglobin (HbA1c), albumin, gamma-glutamyl transferase (γ-GT), total cholesterol, high-density lipoprotein cholesterol (HDL cholesterol), triglycerides, C-reactive protein (CRP), and immunoglobulin E (IgE).

#### 2.4.2. Gut Microbiota

1. DNA Extraction: For DNA extraction, fecal samples in Clinical Virus Transport Medium (Noble Bio Co., Ltd., Seongnam-si, Republic of Korea), 0.1 mm and 0.5 mm glass beads are added. Following agitation, the samples are centrifuged. The supernatant, which is mixed with isopropanol, is applied to a DNA binding column (Bioneer Corporation, Daejeon, Republic of Korea) and centrifuged. Impurities, excluding DNA, were removed using 70% ethanol washing, and pure DNA was extracted using either preheated 65 °C ultrapure water or an extraction buffer.
2. DNA Quantification
  Spectrophotometric Assessment: To assess the purity of the extracted DNA, absorbance measurements were taken at 260 nm, 280 nm, and 230 nm using a NanoDrop spectrophotometer. The A260/A280 ratio was used to estimate protein contamination, with values around 1.8 indicating relatively pure DNA. The A260/A230 ratio was used to evaluate contamination from organic compounds (e.g., phenol, EDTA), with ideal values typically ranging from 2.0 to 2.2. These ratios provided a qualitative measure of DNA purity and were used to screen samples prior to downstream applications.
  Fluorometric Quantification: DNA concentration was determined using the Quant-iT^TM^ PicoGreen^®^ dsDNA Reagent and Kit (Thermo Fisher Scientific, Waltham, MA, USA), which specifically binds to double-stranded DNA. Unlike spectrophotometric methods, PicoGreen is highly sensitive and selective for dsDNA, minimizing interference from RNA or free nucleotides. Fluorescence was measured at excitation/emission wavelengths of approximately 480/520 nm using a microplate reader. DNA concentrations were calculated by interpolating the sample fluorescence values against a standard curve generated from serial dilutions of a DNA standard provided in the kit. This dual approach ensured that only high-quality DNA samples were used for downstream microbiome analyses.
3. PCR Amplicon
  Polymerase Chain Reaction (PCR): PCR was performed using a quantified QC value as a reference. PCR is conducted with an input of greater than 1 ng of DNA, using BX-Taq DNA polymerase (NICSROgene^TM^, Jeonju, Republic of Korea) with a barcode primer set previously designed by the study [11]. The thermal cycling conditions consisted of an initial denaturation at 98 °C for 2 minutes, followed by 30 cycles of 98 °C for 2 min, 50 °C for 30 s, and 72 °C for 150 s, with a final hold at 4 °C. Gel Electrophoresis and Purification: The resulting PCR amplicons were examined for successful generation (4.2 kb) through gel electrophoresis. Magnetic HM SPRI beads (0.45×) are used for bead selection post-PCR. Magnetic purification and 70% ethanol washing were utilized to remove impurities, excluding the amplified PCR amplicons. Pure PCR products were then extracted using preheated ultrapure water or extraction buffer.
  DNA Concentration Adjustment: The final concentration of PCR amplicons was determined using the PicoGreen dsDNA Reagent and Kit, and each sample’s concentration was adjusted to 1000 ng per sample.
4. Pooling and Sequencing: Each PCR amplicon was pooled and processed according to the ligation sequencing amplicon protocol provided by Oxford Nanopore Technology (Oxford, UK, SQK-LSK114, ACDE_9163_v114_revL_29Jun2022). The protocol included end repair, adaptor ligation, clean-up, and loading into flow cells. The third-generation sequencing platform Nanopore was employed to sequence the 16S-ITS-23S operon (rRNA operon).
5. Microbiome Classification and Analysis: Microbiome classification and analysis were conducted using the MIrROR database (http://mirror.egnome.co.kr/) [11] and its associated bioinformatics pipeline, specifically optimized for fecal samples. DNA was amplified using universal rRNA primers, generating a 4.2 kb amplicon to analyze the microbiome composition. To minimize sequencing errors, a custom filtering script was developed to remove false-positive species based on mapping results against reference sequences in the MIrROR database. Filtering criteria included the following:
  Minimum residue-match value, which assesses the similarity between sequencing reads and reference sequences.
  Minimum block-length value, which ensures that a certain length of the read matches the reference sequence consecutively.
6. Further Data Analysis: Statistical analyses were performed using R software (version 4.2.0), utilizing the following R packages: phyloseq, microbiome, ggplot2, vegan, microVIZ, and DESeq2. A custom R function script was also developed to convert read count files (sequence classification results) into an appropriate format for analysis. Microbiome Diversity Analysis: Alpha diversity was assessed using the Chao1 index, Shannon–Wiener index, and Inverse Simpson’s index. Beta diversity, based on the dominant microbiome composition at both phylum and genus levels, was calculated using Bray–Curtis distances and visualized by principal coordinate analysis (PCoA). Statistical significance of differences in microbiome composition between the two groups was evaluated by permutational multivariate analysis of variance (PERMANOVA) with 999 permutations. Additionally, relative abundances at phylum and genus levels were visualized using stacked bar plots. All microbiome analyses were conducted by eGnome, Inc., Seoul, Republic of Korea.

### 2.5. Secondary Outcomes

#### Nutritional Status

Dietary nutrient intake was assessed using the 24 h recall method. To minimize the risk of overestimation of intake, a food frequency questionnaire (FFQ) was not employed. Instead, food and beverage consumption for a single day, including both weekdays and weekends (Saturdays, Sundays, and public holidays), was evaluated through 24 h recall interviews. Nutrient intake was analyzed using the Computer-Aided Nutritional Analysis Program (CAN-PRO 5.0) developed by the Korean Nutrition Society.

### 2.6. Statistical Analysis

The sample size per group was determined based on a previous study examining dietary intervention-induced changes in inflammatory markers [12], resulting in a required sample size of 21 individuals per group. To account for potential dropouts, the final sample size was set at 25 individuals per group. For baseline characteristic comparisons, the Chi-square test was used for categorical variables, while Student’s *t*-test was applied for continuous variables. If the distribution of continuous variables was skewed, the Mann–Whitney U test was used instead. To analyze changes in nutrient intake, blood biomarkers, and gut microbiota composition before and after the intervention, the paired *t*-test was performed. If the differences between pre- and post-intervention values did not follow a normal distribution (as determined by the Shapiro–Wilk test), the Wilcoxon signed-rank test was conducted. The delta values (Δ) between pre- and post-intervention measurements were calculated using the following formula:

Δ (delta values) = post-intervention values – pre-intervention (baseline) values

The between-group differences in delta values were assessed using Student’s *t*-test or the Mann–Whitney U test, depending on data distribution. All statistical analyses were conducted using SPSS 25 (SPSS Inc., Chicago, IL, USA), and a *p*-value < 0.05 was considered statistically significant.

## 3. Results

**Figure 1:**
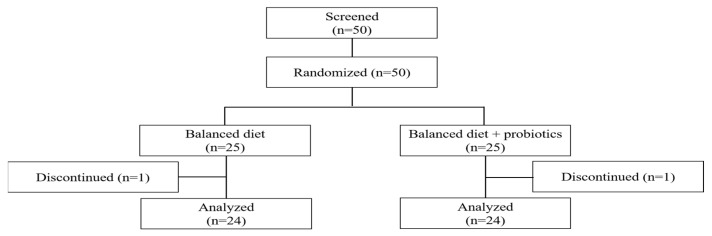
A flowchart showing the study participants who were randomly divided into two groups.

A total of 50 participants who met the inclusion and exclusion criteria were enrolled and randomly assigned to either the B-diet group or the B-diet + probiotics group. Of these, 48 participants completed the trial, with one individual from each group discontinuing participation in the intervention. These individuals were excluded from the final analysis, as shown in the flowchart (Figure 1). The compliance rate with the interventions was greater than 90%. The baseline characteristics of the participants are presented in Table 1. There were no significant differences between the two groups in baseline characteristics except for BMI and blood pressure. The mean BMI was 23.1 ± 2.4 kg/m^2^ in the B-diet group and 25.0 ± 2.5 kg/m^2^ in the B-diet + probiotics group. The mean systolic blood pressure (SBP) was 131.5 ± 23.7 mmHg in the B-diet group and 147.6 ± 22.2 mmHg in the B-diet + probiotics group. The mean diastolic blood pressure (DBP) was 72.0 ± 10.4 mmHg and 78.0 ± 7.3 mmHg in the two groups, respectively.

**Figure 2:**
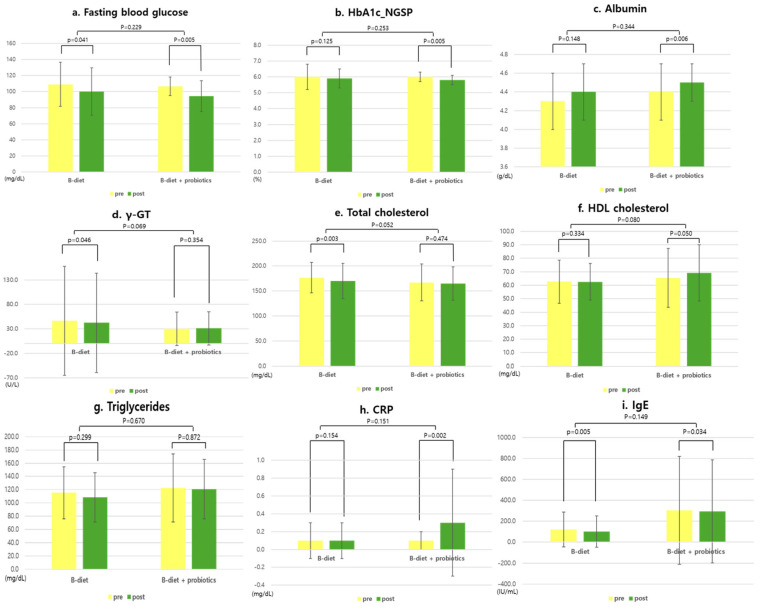
Changes in the biochemical markers before and after the 8-week intervention in each group. Paired *t*-tests or Wilcoxon signed-rank tests were used for within-group comparisons, and Student’s *t*-tests and the Mann–Whitney U tests were used to compare delta values between groups. *p* < 0.05. Abbreviations: HbA1c, hemoglobin A1c or glycated hemoglobin; γ-GT, gamma-glutamyl Transferase; CRP, C-reactive protein; IgE, immunoglobulin E.

Figure 2 presents the changes in blood biomarkers among the primary outcomes at 8 weeks post-baseline. Fasting blood glucose levels significantly decreased in both groups, with no significant difference between the two groups. Significant changes were observed in HbA1c and albumin levels following the intervention in the B-diet + probiotics group (Δ HbA1c −0.2, *p* = 0.005; Δ albumin: +0.1, *p* = 0.006). Significant reductions were observed in γ-GT, total cholesterol, and IgE in the B-diet group: γ-GT decreased from 46.4 ± 111.2 U/L to 42.4 ± 101.2 U/L, total cholesterol decreased from 176.8 ± 30.6 mg/dL to 169.9 ± 35.4 mg/dL, and IgE decreased from 120.2 ± 166.9 IU/mL to 100.1 ± 150.0 IU/mL. Significant changes were observed in CRP and IgE in the B-diet + probiotics group: CRP increased by 0.2 mg/L (*p* = 0.002), and IgE decreased by 9.7 IU/mL (*p* = 0.034). However, there were no significant differences between the two groups in CRP and IgE levels.

**Figure 3:**
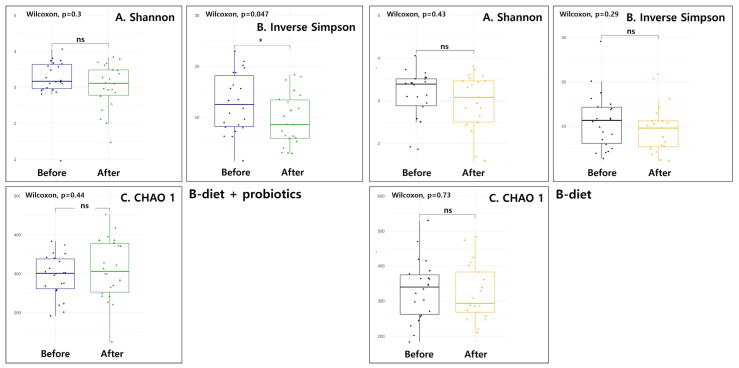
The results of alpha diversity including Shannon–Wiener index, Inverse Simpson’s diversity index, and CHAO 1 diversity index: A Shannon, B Inverse Simpson, C CHAO 1. ns: not significant; *: *p* < 0.05.

Figure 3 presents the phylogenetic alpha diversity indexes, illustrating changes in gut microbial diversity among participants at 8 weeks post-baseline. The Shannon and Chao 1 indexes showed no significant differences after the intervention in either group (Figure 3A,C). However, a significant change was observed in the Inverse Simpson index only in the B-diet + probiotics group (*p* = 0.047), with a lower score post-intervention compared to baseline (Figure 3B). In contrast, no significant change in the Inverse Simpson index was observed in the B-diet group.

**Figure 4:**
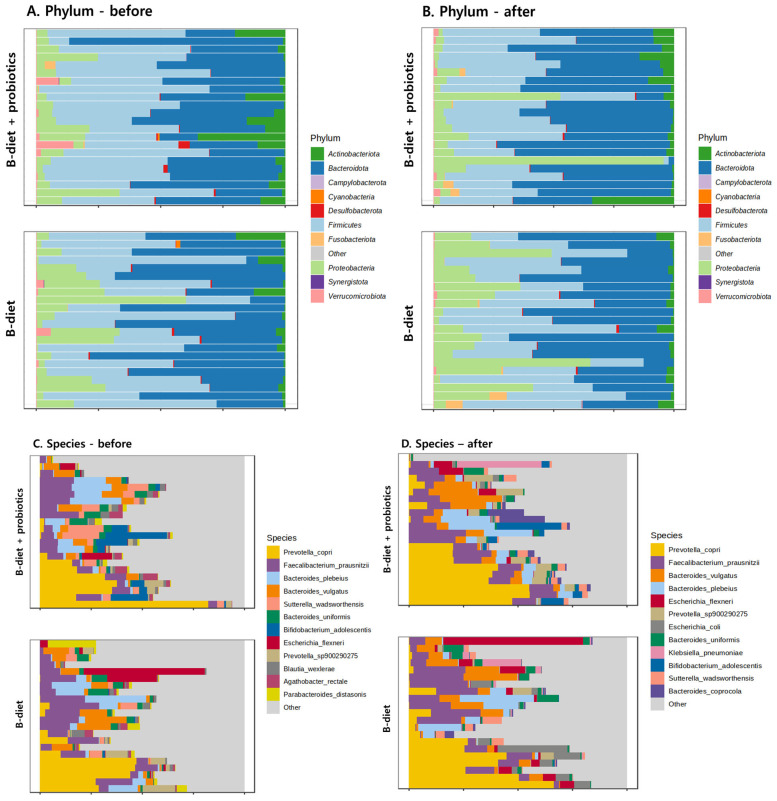
The relative abundance plot of gut microbiota at the phylum and genus levels.

As is consistent with the alterations observed in relative abundances, beta diversity analysis using principal component analysis (PCA) demonstrated distinct microbial profiles between the pre- and post-intervention groups. To further elucidate these differences, the top 12 most prevalent microbes at both the phylum and species levels were identified for each group before and after intervention. At the phylum level, prior to intervention, fecal samples from both groups predominantly exhibited Firmicutes, followed by Bacteroidota (Bacteroidetes). Proteobacteria and Actinobacteriota were present in minor proportions. Post-intervention, however, a marked shift was observed with Bacteroidota becoming dominant in both groups. This shift was particularly pronounced in the B-diet + probiotics group, leading to a decreased Firmicutes/Bacteroidota ratio (F/B ratio). Additionally, the B-diet group experienced a notable increase in the abundance of Proteobacteria post-intervention (Figure 4A,B). At the species level, significant changes were evident in both groups following the intervention. The B-diet group exhibited an increased relative abundance of *Prevotella_copri* and *Faecalibacterium prausnitzii* compared to baseline. Meanwhile, the B-diet + probiotics group demonstrated increases in *Prevotella_copri*, *Faecalibacterium prausnitzii*, *Bacteroides vulgatus*, and *Bacteroides plebeius* relative to pre-intervention levels (Figure 4C,D).

**Figure 5:**
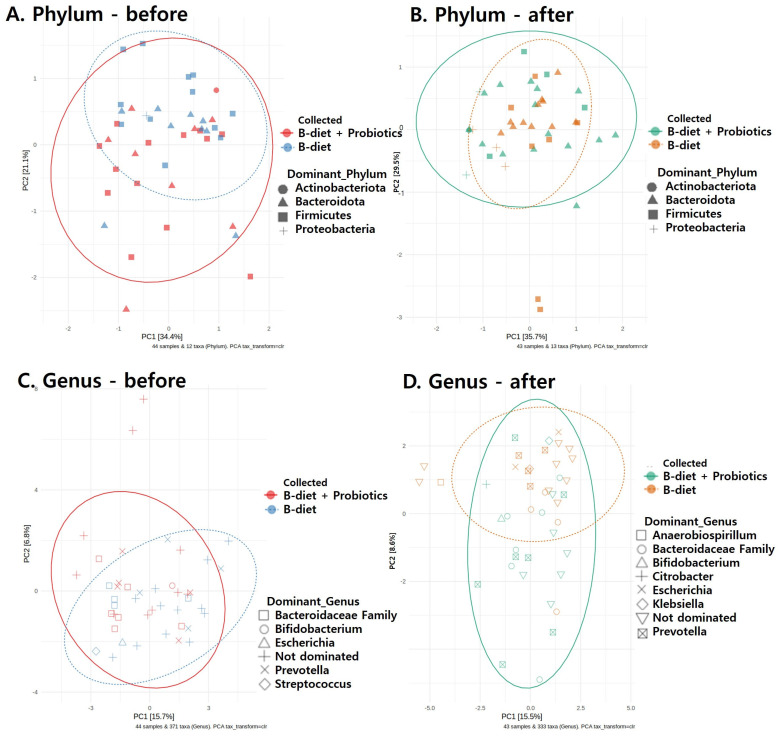
The results of dominant microbiome cluster analysis at the phylum and genus levels.

Figure 5 shows the changes in dominant microbes between the two groups, analyzed at both the phylum and genus levels. At the phylum level, there was little difference in microbiota composition between the B-diet and B-diet + probiotics groups before intervention. After intervention, the microbiome composition in each group shifted in different directions, with a more distinct change observed in the B-diet + probiotics group. Both groups showed a reduction in the F/B ratio, and an increase in Proteobacteria dominance was observed in some samples, with these changes being more pronounced and consistent in the B-diet + probiotics group (Figure 5A,B). At the genus level, following the intervention, the composition of dominant genera became more distinctly separated between the two groups. Notably, the B-diet + probiotics group showed decreased within-group dispersion, indicating increased similarity of microbiota composition among individuals. Furthermore, this group showed sample-specific dominant clusters, notably including the beneficial probiotic genus *Bifidobacterium*. Post-intervention, changes were observed in genera such as *Escherichia*, *Klebsiella*, *Anaerobiospirillum*, and *Citrobacter* in some samples across both groups. In addition, in both groups, samples that were not dominated by a specific genus also formed distinct post-intervention clusters compared to their pre-intervention microbiota composition (Figure 5C,D).

Table 2 shows a comparison of the changes in dietary nutrient intake between the two groups. In both groups, the intake of energy, protein, fat, dietary fiber, vitamin A, vitamin E, vitamin K, folate, calcium, phosphorus, potassium, and iron significantly increased post-intervention compared to pre-intervention. In the B-diet group, the intake of carbohydrates, vitamin D, thiamin, riboflavin, and niacin also significantly increased post-intervention compared to pre-intervention. Significant differences in the delta values between the two groups were observed in the intake of energy, carbohydrate, protein, dietary fiber, vitamin D, thiamin, riboflavin, niacin, folate, phosphorus, potassium, and iron. The carbohydrate-to-protein-to-fat ratio in energy shifted from carbohydrate-dominant to fat-dominant, indicating an improvement. Additionally, in both groups, the proportion of carbohydrate intake significantly decreased post-intervention compared to pre-intervention, while in the B-diet group, the proportion of fat intake significantly increased.

## 4. Discussion

In this study, both the balanced diet combined with probiotics and the balanced diet alone positively influenced the enhancement of blood biomarkers and the gut microbiome in the elderly Korean population. As both groups followed the same balanced diet intervention, with the only difference being the intake of probiotics, within-group improvements were expected, whereas substantial between-group differences were not anticipated. This expectation aligns with the post-intervention findings. However, there were notable baseline imbalances, with the balanced diet + probiotics group having higher BMI, SBP, and DBP. Therefore, these baseline differences should be taken into careful consideration when interpreting the results.

More specifically, when examining diabetes-related biomarkers, although energy and carbohydrate intakes increased, fasting blood glucose and HbA1c significantly improved after the B-diet and probiotics intervention. This outcome suggests that the intervention involving a balanced diet and probiotics is beneficial for managing diabetes in the elderly. On the other hand, in the group following the B-diet alone, a significant improvement was observed only in fasting blood glucose. This aligns with finding from a previously reported study of a similar design, which demonstrated a positive impact on reduced fasting glucose levels in a group adhering solely to a personalized diet [13]. Additionally, a prior study conducted in China showed reductions in fasting glucose and glycated hemoglobin (HbA1c) levels following intensive nutritional management and health education intervention [14]. Furthermore, a meta-analysis of RCTs indicated that high-dose probiotics have an advantageous impact on glycemic homeostasis [15]. Therefore, these results may be attributed to the beneficial impact of increased dietary fiber intake from the balanced diet and probiotics in promoting lower glycemic responses [14,16]. Future research should aim to verify whether these effects are attributable to the balanced diet alone, probiotics alone, or their combined use.

The significant improvement in albumin levels observed in the B-diet + probiotics group is a predictable outcome, given that albumin is an indicator of nutritional status. In contrast, the group receiving only the B-diet did not exhibit a significant enhancement in albumin levels. A previous review highlighted the necessity for further research on the impact of nutritional interventions on albumin level, noting that elderly individuals with low albumin levels often experience significant muscle mass loss and an increased risk of post-surgical complications, including infections [17]. On the other hand, a previous RCT reported that nutritional interventions, such as the inclusion of probiotics, significantly elevated serum albumin levels in malnourished patients undergoing peritoneal dialysis [18]. Thus, the variation in albumin level changes between the B-diet + probiotics group and the B-diet group after intervention may be attributed to the effects of probiotics. Supporting this, a double-blinded RCT that focused on the impact of probiotics on bowel movements in over 80 aged elderly patients found that serum albumin levels significantly increased in the probiotics treatment group compared to the control group [19].

In terms of gamma-glutamyl transferase (γ-GT) levels, the B-diet group showed a significant decrease after the balanced diet intervention. This finding is consistent with a cross-sectional study on Japanese adults, which reported lower γ-GT concentrations in individuals adhering to a healthy dietary pattern [20]. In contrast, no significant change in γ-GT levels was observed in the B-diet + probiotics group. This may be attributed to the fact that their baseline γ-GT levels were already within the normal range, whereas the B-diet group had levels above the normal range. Additionally, participants in the B-diet + probiotics group had higher baseline BMI and blood pressure, indicating a greater degree of metabolic burden that may have attenuated their responsiveness to short-term dietary intervention. To date, limited research has explored the effects of probiotic supplementation or heathy dietary patterns on γ-GT levels, underscoring the need for further studies to evaluate their potential benefits and elucidate the underlying mechanisms. In the B-diet group, an improvement in total cholesterol level was observed following the balanced diet intervention. On the other hand, the B-diet + probiotics group displayed a lower level of total cholesterol, though the change was not statistically significant. This might be due to the baseline cholesterol level being lower than that of the B-diet group. Notably, an increase in high-density lipoprotein (HDL) cholesterol was observed in the B-diet + probiotics group. These findings highlight the beneficial effects of a balanced diet supplemented with probiotics, particularly in the elderly population, and underscore the significance of dietary impacts on lipid profiles. A longitudinal study investigating dietary intakes and plasma lipids in healthy elderly individuals in the United States reported that dietary modifications over a nine-year period led to a significant reduction in fat intake, subsequently influencing cholesterol levels [21]. These results should be interpreted with caution, focusing not merely on fat intake but on overall cholesterol levels. In the US, a healthy diet for the elderly typically involves lower fat intake. Conversely, in South Korea, where diets are predominantly carbohydrate-rich, a healthy diet for the elderly is characterized by lower carbohydrate intake and higher protein and fat intake, within the adequate intake range. Therefore, a balanced diet positively influencing the lipid profiles should be differently designed according to the existing dietary patterns of elderly populations.

With regard to CRP and IgE levels, CRP levels slightly increased by 0.2 mg/L in the B-diet + probiotics group after the 8-week intervention yet remained well within the normal clinical range (<1.0 mg/L). Given the higher baseline BMI and blood pressure in this group, this increase may reflect a low-grade inflammatory state commonly associated with metabolic burden. A previous RCT involving a 56-day personalized diet and probiotic supplementation in healthy older adults similarly reported no significant changes in CRP levels in either the personalized diet (3.6 → 3.8 mg/L) or the diet + probiotics group (2.9 → 2.4 mg/L) [13]. Regarding IgE, both groups in the present study showed reductions following the intervention; however, levels remained above the normal threshold (<100 IU/mL). IgE, an antibody involved in allergic responses, is often elevated in chronic immune conditions. Its production can be influenced by nutritional status, as adequate nutrition supports immune regulation and may help reduce inflammation. Therefore, the observed decrease in IgE may reflect a beneficial impact of the intervention on immune balance, though not sufficient to normalize levels. The persistently elevated IgE concentrations, particularly in the B-diet + probiotics group, may be partly explained by the higher baseline BMI and blood pressure, both of which are linked to enhanced IgE responses and low-grade inflammation. Additionally, age-related immunosenescence, characterized by a shift toward chronic inflammatory activity and reduced capacity to respond to new antigens, may contribute to these findings [6,13,22]. In this context, long-term adherence to a balanced diet rich in anti-inflammatory nutrients such as vitamin E and n-3 polyunsaturated fatty acids may play a key role in modulating immune function in the elderly.

Among the alpha diversity measures addressed, only the Inverse Simpson index showed a significant decrease in the B-diet + probiotics group, suggesting a potential reduction in microbial evenness rather than overall richness. This may indicate the dominance of specific bacterial taxa following the intervention. In contrast, a previous study involving obese elderly women reported an increase in alpha diversity after administration of a balanced hypocaloric Mediterranean diet combined with probiotics [23]. While differences in study design and obesity status may partly explain this discrepancy, baseline differences between the intervention groups in our study, particularly in BMI, SBP, and DBP, which were higher in the B-diet + probiotics group, could also have influenced gut microbial responses. Moreover, variations in dietary fat content between the two studies may have contributed, as prior research has shown that high-fat diets can reduce microbial diversity [24]. Taken together, the observed differences in alpha diversity outcomes may reflect combined effects of baseline host characteristics and diet composition.

When considering the beta diversity analysis and changes in the relative abundance of key microbial taxa, both groups exhibited a significant decrease in the F/B ratio at the phylum level following the intervention. A decreased F/B ratio is well-established to be associated with improved metabolic health; individuals with obesity typically exhibit a higher F/B ratio, and reductions in this ratio following hypocaloric diets or probiotic supplementation suggest a shift toward a metabolically healthier gut microbiota composition [25]. After the intervention, Bateroidota became the predominant phylum in both groups, with a more pronounced shift observed in the B-diet + probiotics group. In contrast, Proteobacteria increased notably in the B-diet group. At the species level, *Faecalibacteium_prausnitzii*, which increased in both groups, showed a greater rise in the B-diet + probiotics group. This species is widely regarded as a biomarker of good gastrointestinal health. In general, *Faecalibacterium*, which tends to decline with aging, contributes to maintaining gut integrity by enhancing the intestinal barrier and exerting anti-inflammatory effects [26]. It plays a key role in gut health through its anti-inflammatory action, support of the gut barrier, and modulation of the immune system [27].

Based on the results of dominant microbiome cluster analysis, increases in *Escherichia* and *Klebsiella* were observed in the B-diet + probiotics group. A previous study in Korea reported that elderly individuals exhibited a higher relative abundance of *Escherichia* compared to younger individuals [28]. Although both *Escherichia* and *Klebsiella* are components of the normal gut flora and play roles in functions such as vitamin synthesis and serve as indicators of gut health, caution is warranted. This is particularly important in elderly populations, as certain strains of these genera possess pathogenic potential and antibiotic resistance, which may negatively affect immune function [29,30]. The observed increase in CRP may reflect a transient immune response or the influence of *Proteobacteria* in certain samples. Nevertheless, in the long term, the dominance of beneficial microbes and a corresponding increase in short-chain fatty acid (SCFA) production may contribute to the stabilization of CRP levels. In the B-diet + probiotics group, the relative abundances of the *Bacteroidaceae* family and *Prevotella* increased following the intervention. *Prevotella* has been reported to decline with age, and its reduction is associated with gut microbiota alterations linked to aging and chronic inflammation [31]. Supporting this, a Korean study of long-lived individuals suggested that a higher abundance of *Prevotella* may be beneficial for promoting longevity and overall health in the elderly [32]. At the species level, *Prevotella_copri* was found to increase post-intervention in both groups. Both *Prevotella_copri* and *Bacteriodes_vulgatus* have been associated with vegetable-rich diets and are recognized as butyrate-producing bacteria, which play important roles in maintaining gut health [33,34]. As people age, physiological changes such as reduced intestinal sensation, impaired motility, and declining immune function can increase vulnerability to gastrointestinal disorders and chronic, low-grade inflammation—a phenomenon often referred to as inflammaging [35]. The present findings suggest that a balanced diet may enrich butyrate-producing gut microbes, which are important for energy metabolism and anti-inflammatory responses. This effect appears to be further enhanced by probiotic supplementation, which positively contributes to the taxonomic profiling and abundance of health-associated microbial taxa in the gut.

The energy intake of the B-diet group and the B-diet + probiotics group, who consumed balanced meals regularly at lunch and dinner, showed significant increases, with 1417.6 kcal and 1433.4 kcal, respectively. At the baseline, both groups did not reach the recommended energy intake for individuals aged 65 and older, as stated in the 2020 KDRIs (men: 2000 kcal; women: 1600 kcal). Such low energy intake levels could impact weight loss, muscle weakness, and slow walking speed, necessitating policies and education to increase energy intake [36,37]. Through the intervention, the nutrient that increased to the recommended intake level was vitamin A. Before the intervention, the B-diet + probiotics group consumed 272.6 µgRAE, but after consuming balanced meals through meal provision, it increased to the level recommended by the 2020 K-DRIs (men: 700 µgRAE; women: 600 µgRAE). Factors such as energy, vitamin A, riboflavin, niacin, and iron intake have been reported to significantly affect the health-related quality of life of elderly individuals in Korea [38,39]. Providing balanced meals to elderly individuals who find meal preparation challenging is considered an essential method for increasing the intake of various nutrients and promoting health improvement.

The traditional Korean dietary pattern is characterized by low meat and high rice consumption, unlike the Western dietary pattern [40]. Similarly to the findings of previous dietary surveys conducted among the Korean elderly, this study also showed a high contribution of carbohydrates to total energy intake [41,42]. In this study, the B-diet group showed a change in the macronutrient distribution (carbohydrates/protein/fat) from 65.9%: 15.0%: 19.2% before the intervention to 59.9%: 15.5%: 24.6% after the intervention. Similarly, the B-diet + probiotics group showed a decrease in the contribution of carbohydrates from 59.3%: 15.2%: 25.5% at baseline to 56.0%: 15.9%: 28.1% after the intervention. These results indicate that consuming balanced meals led to a reduced proportion of energy derived from carbohydrates. However, regarding the relative contribution of fat to total energy intake, the traditional Korean diet characterized by high carbohydrates and low protein and fat content may make the current balanced diet for older Korean adults appear to have a relatively higher fat proportion. As the dietary patterns of the next generation differ from those of the current older generation, the ideal macronutrient composition of a balanced diet may need to be adjusted substantially, especially in light of today’s rapidly changing dietary trends. Consequently, caution is warranted when interpreting these findings. As noted earlier, balanced dietary interventions aimed at improving lipid profiles should be tailored to the prevailing dietary habits of the specific elderly population under study.

Through the community-based intervention study of a balanced diet tailored to address the chewing and swallowing difficulties of the elderly, along with probiotics intervention, improvements in blood biomarkers, gut microbiota, and nutrient intake among the elderly were observed. The results of this study suggest the necessity of intervention aimed at enhancing immunity to improve the health of the elderly. Based on this, it is expected that active nutritional education and policy exploration and implementation regarding balanced diets for the elderly will contribute to improving their health and quality of life. However, the present study has several limitations, including its short duration, small sample size, absence of a proper control group (both a no-intervention control and a probiotics-only control), uneven sex distribution, and potential performance bias due to the single-blinded design. Furthermore, despite random group assignment, the B-diet + probiotics group had higher baseline BMI and blood pressure, which may have affected the observed differences in outcomes between the two intervention groups.

## 5. Conclusions

To the best of our knowledge, this study is the first to investigate the effects of a balanced diet alone and in combination with probiotics on blood parameters and gut microbiota composition in elderly Koreans. The main finding of this 8-week intervention study is that a balanced dietary intervention beneficially impacts nutritional and inflammatory biomarkers, including fasting glucose, HbA1c, albumin, γ-GT, total cholesterol, HDL cholesterol, and IgE, as well as gut microbiota composition. Notably, the balanced diet resulted in favorable microbiota changes, characterized by a reduction in the F/B ratio and increased abundances of the *Bacteroidaceae* family, as well as *Prevotella* and *Faecalibacterium* at the genus level. Furthermore, probiotic supplementation enhanced these beneficial effects, suggesting a synergistic or complementary impact on the composition and abundance of health-associated gut microbiota. These results indicate that combining a balanced dietary approach with probiotics could be an effective strategy to improve gut microbiota health and associated metabolic markers in older adults. However, future RCTs employing longer durations, larger sample sizes, and double-blinded designs are necessary to validate these findings and elucidate underlying mechanisms.

## Figures and Tables

**Table 1:** Baseline characteristics of participants.

Variables	B-Diet (n = 24)	B-Diet + Probiotics (n = 24)	*p*-Value ^1^
Age, y	81.5 (9.5)	82.2 (5.5)	0.77
Sex, female, %	62.5	87.5	0.05
Weight, kg	55.2 (8.1)	57.9 (7.0)	0.24
BMI, kg/m^2^	23.1 (2.4)	25.0 (2.5)	0.01
SBP, mmHg	131.5 (23.7)	147.6 (22.2)	0.02
DBP, mmHg	72.0 (10.4)	78.0 (7.3)	0.02
Educational level, %			0.78
≤Elementary	30.4	41.7	
Middle school	4.3	20.8	
High school	21.7	20.8	
College or university	43.5	12.5	
≥Graduate school	0.0	4.2	
Stress status, %			0.91
No	41.7	45.8	
Mild to Moderate	50.0	41.7	
Severe	4.2	8.3	
Very severe	4.2	4.2	
Sleep duration, h	6.9 (1.4)	6.6 (1.9)	0.40
Alcohol intake, %			0.38
None	65.2	79.2	
Past drinkers	8.7	0.0	
Current drinkers	26.1	20.8	
Smoking status, %			0.22
None	82.6	95.8	
Past smokers	13.0	4.2	
Current smokers	4.3	0.0	
Regular exercise			0.16
Yes	87.5	70.8	
No	12.5	29.2	
K-IADL	9.8 (2.7)	11.6 (4.8)	0.17

Data are presented as the percentage of participants or mean ± SD. Abbreviations: B-diet, balanced diet; BMI, body mass index; SBP, systolic blood pressure; DBP, diastolic blood pressure; K-IADL, K-instrumental activities of daily living. ^1^ Differences between groups were evaluated by the Chi-square test, Mann–Whitney U test, and Student’s *t*-test.

**Table 2:** Comparison of changes in dietary nutrient intake between groups.

Variables	B-Diet			B-Diet + Probiotics			Comparison of Δ Values Between Groups ^2^
	Pre	Post	*p*-Value ^1^	Pre	Post	*p*-Value ^1^	
Energy (kcal)	989.9 (279.5)	1433.4 (312.4)	0.00	1247.8 (375.4)	1417.6 (271.9)	0.01	0.01
Carbohydrate (g)	161.4 (42.3)	211.6 (43.8)	0.00	184.1 (65.5)	196.6 (37.4)	0.05	0.01
Protein (g)	37.1 (13.7)	55.6 (14.8)	0.00	47.0 (14.1)	55.2 (9.3)	0.01	0.02
Fat (g)	21.5 (11.1)	39.4 (11.2)	0.00	34.4 (13.2)	44.6 (14.0)	0.00	0.07
Carbohydrate (%)	65.9 (8.1)	59.9 (5.6)	0.01	59.5 (7.6)	56.0 (5.1)	0.05	0.36
Protein (%)	15.0 (2.6)	15.5 (2.0)	0.46	15.3 (2.1)	15.9 (2.0)	0.35	0.96
Fat (%)	19.2 (6.2)	24.6 (3.9)	0.00	25.2 (6.6)	28.1 (5.9)	0.08	0.26
Dietary fiber (g)	14.7 (5.7)	23.4 (7.3)	0.00	18.3 (5.6)	23.0 (3.6)	0.00	0.02
Vitamin A (µgRAE)	182.5 (81.3)	571.0 (241.5)	0.00	270.6 (188.3)	720.8 (116.4)	0.00	0.37
Vitamin D (µg)	1.1 (1.3)	3.1 (2.6)	0.00	2.1 (2.1)	2.1 (0.9)	0.98	0.03
Vitamin E (mg)	7.2 (3.4)	19.1 (6.2)	0.00	10.8 (4.2)	20.7 (4.9)	0.00	0.22
Vitamin K (µg)	87.0 (85.5)	269.5 (167.7)	0.00	100.1 (93.0)	283.6 (77.7)	0.00	0.98
Vitamin C (mg)	61.0 (37.1)	70.8 (55.6)	0.42	38.8 (36.2)	68.5 (36.0)	0.98	0.52
Thiamin (mg)	1.0 (0.3)	1.4 (0.3)	0.00	1.3 (0.4)	1.3 (0.3)	0.97	0.00
Riboflavin (mg)	0.7 (0.4)	1.2 (0.4)	0.00	1.1 (0.5)	1.3 (0.4)	0.11	0.02
Niacin (mg)	6.5 (2.6)	10.3 (2.8)	0.00	8.9 (3.1)	10.1(1.6)	0.05	0.00
Folate (µgDFE)	269.5 (96.8)	470.4 (133.6)	0.00	345.9 (120.1)	450.4 (87.1)	0.00	0.01
Calcium (mg)	296.6 (96.7)	536.6 (184.6)	0.00	422.5 (199.2)	555.6 (113.9)	0.01	0.09
Phosphorus (mg)	637.4 (200.9)	927.1 (242.6)	0.00	771.7 (209.3)	925.3 (172.8)	0.01	0.03
Sodium (mg)	2245.3 (906.0)	3908.9 (1089.4)	0.00	2109.9 (760.2)	3617.3 (653.3)	0.00	0.57
Potassium (mg)	1614.8 (524.6)	2826.9 (756.3)	0.00	1884.3 (495.4)	2658.1 (542.1)	0.00	0.02
Magnesium (mg)	76.8 (40.2)	76.6 (45.0)	0.99	69.3 (37.3)	58.4 (25.3)	0.15	0.41
Iron (mg)	8.1 (2.5)	16.2 (10.5)	0.03	10.4 (3.2)	14.2 (2.7)	0.00	0.02

Data are presented as mean (SD). ^1^ Paired *t*-tests and Wilcoxon signed-rank tests were applied to examine changes between interventions. ^2^ Differences in delta values between groups were assessed using Student’s *t*-test.

## Data Availability

The original contributions presented in this study are included in the article. Further inquiries can be directed to the corresponding author.
